# Corrigendum: Accessing care services after sexual violence: A systematic review exploring experiences of women in South Africa

**DOI:** 10.4102/curationis.v47i1.2576

**Published:** 2024-01-10

**Authors:** Moreoagae B. Randa, Julie McGarry, Sarah Griffiths, Kathryn Hinsliff-Smith

**Affiliations:** 1 Department of Public Health, Faculty of Health Care Sciences, Sefako Makgatho Health Sciences University, Pretoria, South Africa; 2 Health Sciences School, University of Sheffield and Sheffield Teaching Hospitals NHS Foundation Trust, Sheffield, United Kingdom; 3 Leicester School of Nursing and Midwifery, Faculty of Health and Life Sciences, De Montfort University, Leicester, United Kingdom

In the published article, Randa, M.B., McGarry, J., Griffiths, S. & Hinsliff-Smith, K., 2023, ‘Accessing care services after sexual violence: A systematic review exploring experiences of women in South Africa’, *Curationis* 46(1), a2405. https://doi.org/10.4102/curationis.v46i1.2405. The authors neglected to include the funder South African Medical Research Council, SIR:058 in the funding statement.

The authors apologise for this error. The correction does not change the study’s findings of significance or overall interpretation of the study’s results or the scientific conclusions of the article in any way.

